# Molecular and cytogenetic analysis of small supernumerary marker chromosomes in prenatal diagnosis

**DOI:** 10.1186/s13039-023-00655-z

**Published:** 2023-09-04

**Authors:** Yang Yang, Wang Hao

**Affiliations:** 1 Prenatal Diagnosis Center, Hangzhou Maternity and Child Care Hospital, #369 Kunpeng Road, Shangcheng District, Hangzhou, 310008 Zhejiang China; 2 Department of Cell Biology and Medical Genetics, School of Medicine, Zhejiang University, Hangzhou, Zhejiang China

**Keywords:** Small supernumerary marker chromosome, Prenatal diagnosis, Chromosomal microarray analysis

## Abstract

**Background:**

Small supernumerary marker chromosome (sSMC) is a structurally abnormal chromosome of unknown origin by conventional cytogenetics. The understanding of clinical significance of sSMC is still limited in prenatal diagnosis. The presence of sSMC poses a challenge for genetic counselling.

**Methods:**

We obtained the clinical information of 25 cases with sSMC. The fetal samples were subjected to multiple molecular and cytogenetic approaches including karyotype analysis, chromosomal microarray analysis, bacterial artificial chromosomes-on-beads assay, and fluorescence in situ hybridization.

**Results:**

Seven sSMCs were found to be r(X), and five of the cases terminated the pregnancy. Three markers were idic(15), and one of the cases was normal at birth. Two markers were i(12p), and both cases terminated the pregnancy. Other markers were r(Y) (outcome: normal at birth), i(18p) (outcome: stillbirth), der(15) (outcome: terminated), del(9) (outcome: terminated), dup(13) (outcome: follow-up loss), and derived from chromosome 21 (outcome: stillbirth). Seven markers were of unknown origin because not all methods were applied to them.

**Conclusion:**

Applying multiple molecular and cytogenetic approaches could identify the origin and genetic content of sSMC to assist the genetic counselling in prenatal diagnosis.

## Introduction

Marker chromosomes are defined as structurally abnormal chromosomes of unknown origin by conventional cytogenetics. Small supernumerary marker chromosome (sSMC), as a common form of marker chromosomes, can be present in addition to 46,XX or 46,XY or numerically abnormal karyotypes such as Turner syndrome [1]. In the prenatal setting, the prevalence of sSMCs is estimated at 0.075% [2]. Most sSMCs are de novo and originated from acrocentric chromosomes [3].

The clinical consequences caused by sSMCs vary significantly, making it challenging for the prenatal genetic counseling [4]. With the wide application of chromosomal microarray analysis in prenatal diagnosis, the origins and genetic contents of sSMCs could be revealed, which would expanding our understanding of the clinical significance of sSMCs.

In this study, we used multiple molecular and cytogenetic approaches to investigate 25 sSMCs cases to identify the origins and genetic compositions of the markers and to better understand the genotype–phenotype relationship of sSMCs.

## Materials and methods

### Subjects

We retrospectively reviewed the sSMCs detected in our center from 2016 to 2022. 25 cases were found to have sSMCs (Table 1). The pregnant women of the cases received either transabdominal amniocentesis or cordocentesis, and the fetal specimens were obtained. All patients gave their informed consent prior to their inclusion in the study. This study was approved by the Ethics Committee of Hangzhou Maternity and Child Care Hospital.

**Table 1:** Clinical information and karyotypes of the cases with sSMCs

No.	Age	Indication	Gestational age (weeks)	Specimen	Karyotype	Outcome
1	27	MSS abnormality	21	Amniotic fluid	47,XX, + mar	Normal at birth
2	32	NIPT abnormality (chromosome X)	22	Amniotic fluid	mos 46,X,r(X)[32]/45,X[18]	Terminated
3	27	MSS abnormality	20	Amniotic fluid	47,XY, + dup(13)(q11q12.12)	Unknown
4	39	NIPT abnormality (chromosome X)	19	Amniotic fluid	47,XX, + mar	Unknown
5	42	AMA	19	Amniotic fluid	mos 47,XY, + i(12)(p10)[14]/46,XY[16]	Terminated
6	37	AMA	19	Amniotic fluid	47,XY, + idic(15)(q13.3)	Unknown
7	27	MSS abnormality	19	Amniotic fluid	mos 46,X,r(X)(p11.22q21.1)[36]/45,X[14]	Unknown
8	40	AMA	22	Amniotic fluid	47,XX, + mar	Terminated
9	41	AMA	20	Amniotic fluid	47,XX, + der(15)t(6;15)(p25.3;q13.2)	Terminated
10	35	MSS abnormality	20	Amniotic fluid	mos 47,XY, + mar[14]/46,XY[36]	Normal at birth
11	25	NIPT abnormality (chromosome X)	19	Amniotic fluid	46,X,r(X)(q11.1q21.1)	Terminated
12	27	NIPT abnormality (chromosome X)	21	Amniotic fluid	mos 47,XX, + r(X)(p11.21q13.1)[27]/46,X,r(X)(p11.21q13.1)[1]/46,XX[8]	Terminated
13	30	Adverse pregnancy history	18	Amniotic fluid	47,XX, + idic(15)(q13.3)	Normal at birth
14	38	AMA, MSS abnormality	19	Amniotic fluid	mos 47,XX, + mar[3]/46,XX[29]	Normal at birth
15	40	AMA, ultrasound abnormality	24	Amniotic fluid	mos 47,XY, + i(12)(p10)[10]/46,XY[18]	Terminated
16	29	Adverse pregnancy history	19	Amniotic fluid	mos 47,XX, + mar[3]/46,XX[36]	Normal at birth
17	27	Ultrasound abnormality	25	Amniotic fluid	mos 45,X[33]/46,X,r(X)(p11.21q21.1)[18]	Terminated
18	36	MSS abnormality	22	Amniotic fluid	mos 47,XY, + der(21)[12]/46,XY[84]	Stillbirth
19	26	NIPT abnormality (chromosome 9)	15	Amniotic fluid	mos 47,XX, + 9[12]/47,XX, + mar[5]/46,XX[33]	Unknown
20	35	AMA, NIPT abnormality (chromosome 15)	14	Amniotic fluid	47,XY, + idic(15)(q13.3)	Unknown
21	36	AMA, ultrasound abnormality	26	Cord blood	mos 46,X,r(X)(p11.1q13.3)[35]/45,X[15]	Terminated
22	34	Ultrasound abnormality	27	Cord blood	mos 45,X[26]/46,X,r(X)(p11.3q13.3)[24]	Unknown
23	29	Ultrasound abnormality	25	Cord blood	47,XY, + del(9)(q12)	Terminated
24	39	AMA	26	Cord blood	mos 47,XX, + i(18)(p10)[5]/46,XX[47]	Stillbirth
25	27	NIPT abnormality (chromosome X)	27	Cord blood	mos 46,X,r(Y)[16]/45,X[4]	Normal at birth

*AMA* advanced maternal age, *NIPT* non-invasive prenatal test, *MSS* maternal serum screening

### Karyotyping

Transabdominal amniocentesis and cordocentesis were conducted under sterile circumstances according to standard procedures. Cultured amniotic fluid cells and cord blood lymphocytes were harvested and subjected to G-band staining. The karyotypes were described according to the International System for Human Cytogenomics Nomenclature 2020 (ISCN2020) [5].

### Chromosomal microarray analysis

CMA was applied to the fetal specimens using Affymetrix CytoScan 750K arrays (Affymetrix, CA, USA) according to the manufacturer's instructions. Data analysis was performed by Chromosome Analysis Suite software (Affymetrix, CA, USA) to identify clinically significant copy number variants (CNVs). The pathogenicity of detected CNVs was assessed in accordance with the technical standards issued by the American College of Medical Genetics and Genomics and the Clinical Genome Resource (ClinGen) [6].

### Bacterial artificial chromosomes-on-beads (BoBs) assay

BoBs assay was performed following the manufacturer’s protocol (PerkinElmer, MA, USA). The data was analyzed usingBoBsoft1.1 rev 2 software (PerkinElmer, MA, USA).

### Fluorescence in situ hybridization (FISH)

FISH analysis was carried out according to the manufacturer’s instructions (Vysis; Abbott Molecular, IL, USA). The probe for Case 2 was SRY/DXZ1 probe, which was specific for the sex determining region of Yp11.3 and alpha satellite DNA of Xp11.1-q11.1. The probe for Case 5 was specific for 12pter/12qter. The 13/21 probe for Case 18 was used to detect 13q14 (RB1) and 21q22.13-21q22.2 region (D21S259/D21S341/D21S342).

## Results

Clinical information and karyotypes of the cases were listed in Table 1. Fifteen of them were mosaics, and the mosaic ratio ranged from 7.69 to 80%. Eight sSMCs were accompanied by additional chromosomal number abnormalities (seven Turner syndrome cases and one chromosome 9 trisomy case).

**Fig. 1:**
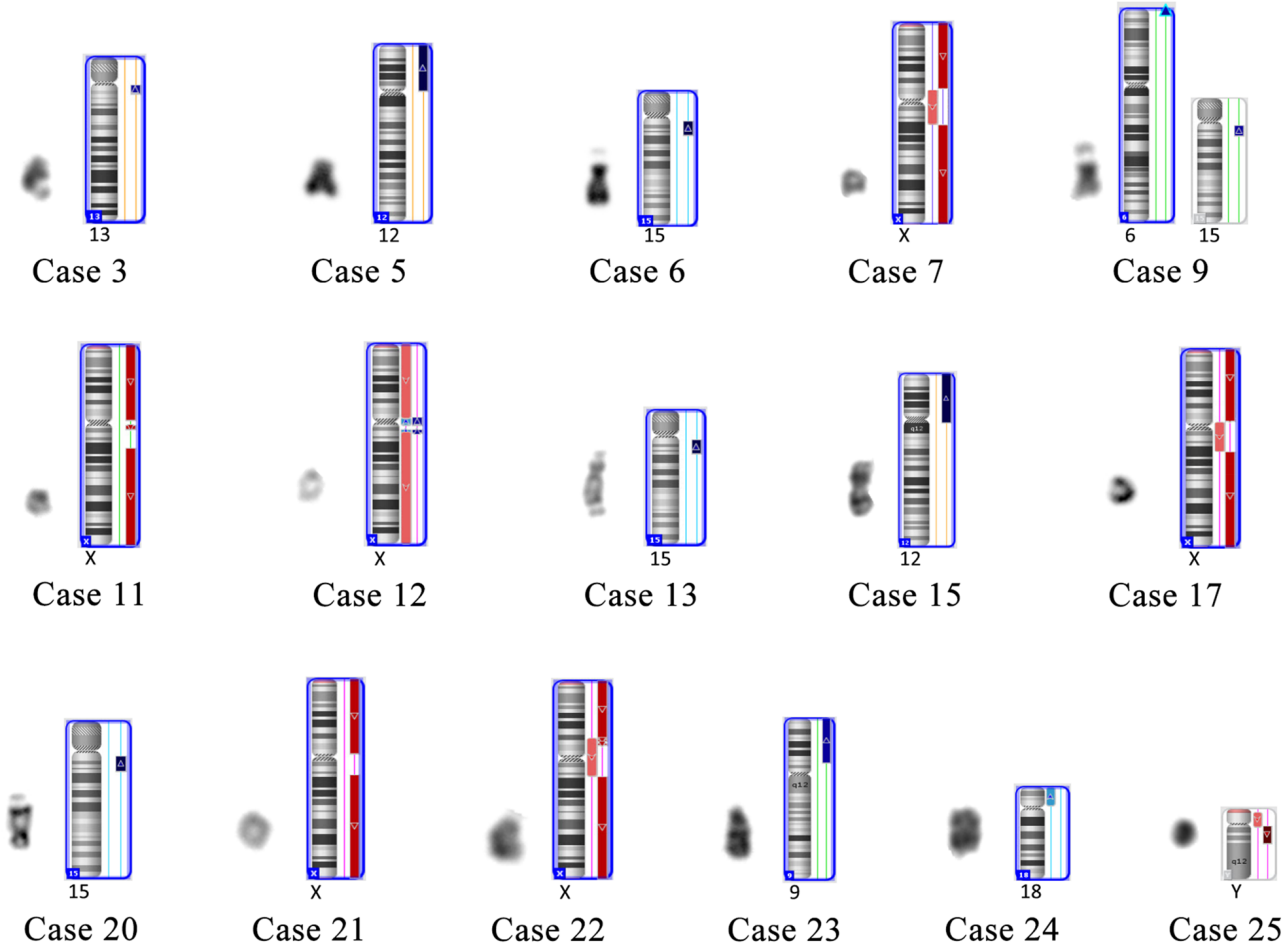
The morphology and idiograms of sSMCs with abnormal CMA results. Case 3: arr[hg19] 13q11q12.12(19,436,286–25,319,733) × 3; Case 5: arr[hg19] 12p13.33p11.1(173,786–34,835,641) × 4; Case 6: arr[hg19] 15q11.2q13.3(22,770,421-32,915,723) × 4; Case 7: arr[hg19] Xp22.33p11.22(168,551-52,877,319) × 1,Xp11.22q21.1(52,877,320–77,345,323) × 1–2,Xq21.1q28(77,345,324-155,233,098) × 1; Case 9: arr[hg19] 6p25.3(156,974–889,487) × 3,15q11.2q13.2(22,770,421–30,913,574) × 3; Case 11: arr[hg19] Xp22.33q11.1(168,551–62,051,248) × 1,Xq11.2q12(63,002,581–65,354,853) × 1,Xq21.1q28(80,602,173-155,233,098) × 1; Case 12: arr[hg19] Xp22.33p11.21(168,551-57,677,734) × 1–2,Xp11.21q11.2(57,677,735–63,489,866) × 2–3,Xq12q13.1(67,062,445-68,992,710) × 2-3,Xq13.1q28(68,992,711-155,233,098) × 1–2; Case 13: arr[hg19] 15q11.2q13.3(22,770,421-32,915,723) × 4; Case 15: arr[hg19] 12p13.33q11(173,786–37,858,351) × 4; Case 17: arr[hg19] Xp22.33p11.21(168,551_58,527,154) × 1,Xp11.21q21.1(58,527,155_80,103,965) × 1–2, Xq21.1q28(80,103,966_155,233,098) × 1; Case 20: arr[hg19] 15q11.2q13.3(22,770,422_32,444,261) × 4; Case 21: arr[hg19] Xp22.33p11.1(168,551–58,112,823) × 1,Xq13.3q28(75,024,481–155,233,098) × 1; Case 22: arr[hg19] Xp22.33p11.3(168,551–45,446,616) × 1,Xp11.3q13.3(45,518,515–75,045,175) × 1–2,Xq13.3q28(75,404,909–155,233,098) × 1; Case 23: arr[hg19] 9p24.3p13.1(208,454–38,787,480) × 3; Case 24: arr[hg19] 18p11.32p11.21(136,227–15,181,208) × 2–3; Case 25: arr[hg19] Yp11.31q11.21(2,650,424–14,736,209) × 0–1,Yq11.21q11.23(14,736,210–28,799,654) × 0

CMA and BoBs assay were conducted to investigate the genetic composition of the sSMCs. 24 cases underwent CMA, and only Case 2 was subjected to BoBs assay. The BoBs assay result of Case 2 indicated the presence of Xq13 and the deletion of Xp22, Xp21 and Xq27. The CMA results of Case 1, 4, 8, 10, 14, 16, 18 were normal. Case 1, 4, and 8 were non-mosaic, suggesting that these markers would probably originate from the heterochromatin region which the probes of CMA did not cover. Case 10, 14, 16, and 18 were mosaic. Therefore, the low proportion mosaic could also lead to the normal results of CMA. Abnormal CMA results were displayed in Fig. 1.

According to the CMA results and karyotypes, the markers of Case 7, 11, 12, 17, 21, and 22 were most likely to be r(X). The marker of Case 25 was inferred to be r(Y). The markers of Case 6, 13, 20 were supposed to be idic(15). The markers of Case 5 and 15 were inferred to be i(12p). The marker of Case 24 was likely to be i(18p). Because the father of the fetus in Case 9 was a carrier of balanced translocation: 46,XY,t (6;15)(p25.3;q13.2), the marker of Case 9 was probably der(15) originating from the translocation of chromosome 6 and 15. The markers of Case 3 and 23 might be part of chromosome 13 and 9, respectively. In Case 19, the low mosaic proportion of the marker and the coexistence of mosaic trisomy 9 made it difficult to infer the origin of the marker.

**Fig. 2:**
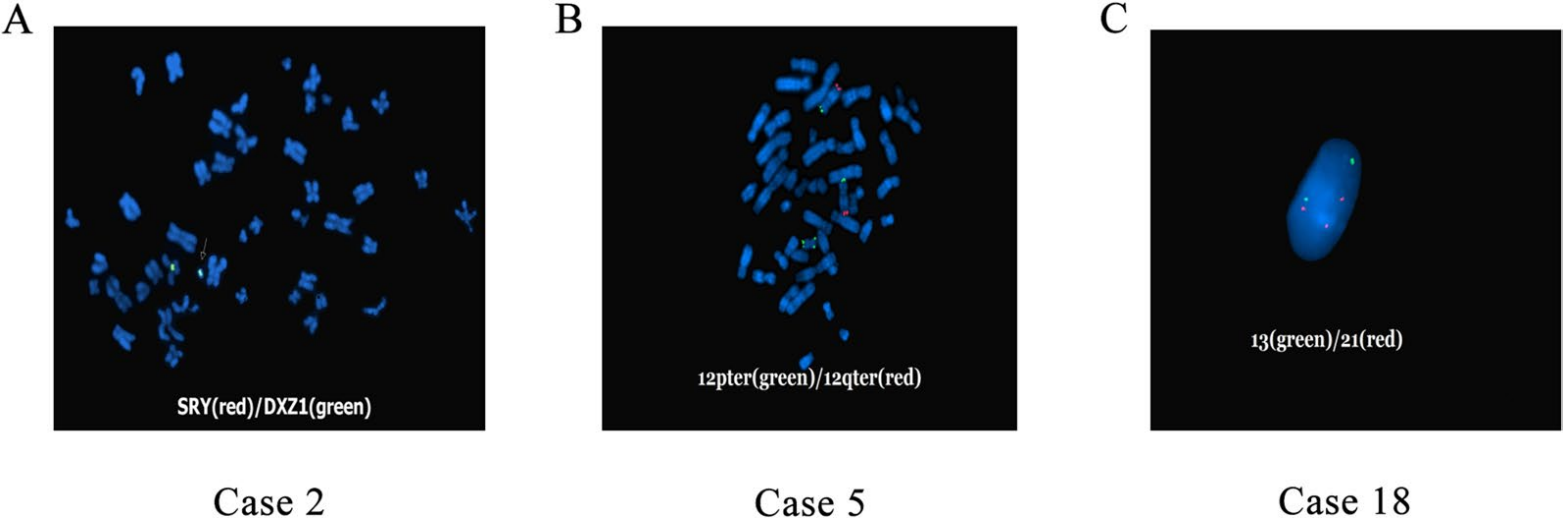
The FISH results of Case 2, 5, and 18. **A** In Case 2, two DXZ1 signals were observed, and one signal was found at the sSMC. **B** In Case 5, two chromosome 12 showed normal 12pter/12qter signals, and two additional 12pter signals were observed at the sSMC. **C** In Case 18, three signals specific for 21q22.13-21q22.2 region were observed

FISH was used to validate the origin of the markers of Case 2, 5, and 18 (Fig. 2). We concluded that the marker of Case 2 was r(X), and the marker of Case 18 originated from chromosome 21. The marker of Case 5 was confirmed to be i(12)(p10).

Because FISH was not applied to all samples, 7 markers of the study were still of unknown origin. Three of them were non-mosaic, and the clinical outcomes varied. Four of them were mosaic, and the clinical outcomes were normal at birth except that Case 19 with mosaic trisomy 9 was lost to follow-up.

## Discussion

Although low proportion mosaic, coexistence of other unbalanced chromosomal rearrangement, and the probe distribution of CMA would affect the detection of the origin and genetic content of sSMC, CMA could reveal the genetic compositions of most sSMCs. But CMA could not identify the regions that the probes of CMA do not cover, such as the centromere. Under this circumstance, FISH is vital for the identification of the centromere. Therefore, it is important to apply multiple molecular and cytogenetic approaches to fully understand the origin and genetic content of sSMC.

In the present study, r(X) was frequently observed, and it was often present with 45, X. The phenotypes of turner syndrome patients with r(X) have been reported to be severe, including growth retardation, intellectual disability, and multiple malformations [7–9]. The loss of X-inactive specific transcript (XIST) at Xq13 leads to functional disomy of the proximal region of chromosome X, causing the severe phenotypes [1, 10]. The proportion of cells with 45, X could also affect the severity of phenotypes [11]. Case 17 was found to have cerebellar hypoplasia, and Case 22 had hypoplastic left heart. The other cases with r(X) in our study did not show significant abnormalities in prenatal ultrasound scanning. This is probably because the proportions of cells with 45, X in Case 17 and Case 22 were both more than 50%, and most of the other cases with r(X) in the present study had low-level mosaicism of 45,X and contained XIST at Xq13. Other abnormalities such as amenorrhea and development retardation need long-term follow up, but many families in the study chose to terminate the pregnancy. Three cases were found to have idic(15). The clinical consequence of cases with idic(15) is associated with the parental origin and the presence and dosage of the Prader–Willi/Angelman syndrome critical region (PWACR) [1, 12]. The cases with idic(15) derived from mother and father differ in phenotype, but they also have some overlapping phenotypic features [13]. In the present study, the three cases with idic(15) all included PWACR, but only Case 13 had a normal outcome at birth. The other two cases were lost to follow up. Abnormal phenotypes would probably arise along with the growth and development of the child. As shown in ChromosOmics Database (https://cs-tl.de/DB/CA/sSMC/0-Start.html) [accessed on 2023/7/23], many cases with idic(15) had no clinical findings. Mental symptoms were common in cases with idic(15), which would be identified at a certain age. Two markers of our study were i(12p). Cases with i(12p) are known to be associated with Pallister–Killian syndrome, which has highly variable phenotypes involving multiple systems [14]. Facial dysmorphism, developmental delay, mental retardation, and hypotonia were observed in all i(12p) cases in ChromosOmics database. However, clinical information that we could obtain from the two prenatal cases were limited. The ultrasound result of Case 5 was normal, and the ultrasound of Case 15 showed the dilatation of bilateral lateral ventricles and echogenic intracardiac focus. The two cases were both mosaic i(12p), and researchers found that even low proportion mosaic i(12p) would probably lead to abnormal clinical outcome [4]. One case of the present study had i(18p). No abnormality was found in the ultrasound of Case 24. Isochromosome 18p syndrome would cause growth and mental retardation, neonatal respiratory distress, and characteristic dysmorphism [15, 16]. Case 25 was found to have r(Y). The appearance of patients with the presence of 45, X and r(Y) could be male or female [17]. The phenotypic sex is related to the SRY gene copy number, and the variable degree of mosaicism in different tissues would affect the phenotype [18, 19]. The marker of Case 9 was der(15) inherited from the father. The karyotype was non-mosaic. Therefore, the non-disjunction of chromosome 15 was likely to occur in meiosis. The CNV of chromosome 6 was identified as variant of uncertain significance, while the CNV of chromosome 15 was identified to be pathogenic because it involves PWACR. The marker of Case 23 was derived from chromosome 9, leading to trisomy 9p. Trisomy 9p has been reported in more than 150 cases and has been well recognized [20]. The phenotypic features of trisomy 9p are variable including microbrachycephaly, growth and mental retardation, hand and foot malformations, and dysmorphic features [21, 22]. The prenatal information is limited. In this case, the dilatation of bilateral lateral ventricles and absent nasal bone were observed through ultrasound.

In our study, ten cases terminated the pregnancy. Case 18 and 24 were stillbirth. In Case 18, the CMA result was normal, but a second extraction and karyotyping of the amniotic fluid confirmed the existence of the mosaic marker. FISH result demonstrated that the marker is derived from chromosome 21, leading to partial trisomy 21. The cytogenic location of the FISH probe is 21q22.13-q22.2, involving Down syndrome critical region [23]. Considering that the mosaic proportion was low, the marker might have a strong impact on the growth and development of the fetus. The CMA result of Case 24 demonstrated that the CNV was pathogenic. The duplication contains 54 OMIM genes, and it has been reported that tetrasomy18p would cause developmental delay and intellectual disability [15, 16]. Six cased continued the pregnancy and the babies were phenotypically normal at birth. Three of them were low proportion mosaic and demonstrated normal CMA results. Case 1 was normal at birth indicating that the heterochromatin marker would not impact the phenotype of the baby. The CMA result of Case 13 showed pathogenic CNV. The duplication involves PWACR, which is related to 15q duplication syndrome and the clinical manifestation includes varying degrees of hypotonia, intellectual disability, autism spectrum disorder, and epilepsy [24–26]. Some symptoms would probably not arise at birth, but could be late-onset [27]. The CNVs found in Case 25 were also pathogenic. There was a suspected low proportion mosaic deletion of Yp11.31q11.21 and a deletion of Yq11.21q11.23 segment. The deletion regions encompass SRY gene and AZF loci, which would hinder the sexual development and spermatogenesis of the child in the future [28, 29].

In conclusion, the phenotypes of sSMCs are highly variable. Though some sSMCs are known to be associated with specific syndromes, there are overlaps in phenotypic features. In addition, the presence of mosaic and other chromosomal imbalances, uniparental disomy, and the parental origin would also affect the clinical consequences of sSMCs [3]. Thus, it is difficult to precisely predict the genotype–phenotype correlation. Generally speaking, the risk for acrocentric sSMCs is low, and the risk for nonacrocentric sSMCs is higher [4]. We need to use multiple technologies to find out the origin and genetic content of the marker to evaluate the risk for each sSMC.

## Data Availability

The datasets used and/or analyzed during the current study are available from the corresponding author on reasonable request.
